# Survival of highly related ESBL- and pAmpC- producing *Escherichia coli* in broiler farms identified before and after cleaning and disinfection using cgMLST

**DOI:** 10.1186/s12866-024-03292-7

**Published:** 2024-04-25

**Authors:** Caroline Robé, Michaela Projahn, Katrin Boll, Anja Blasse, Roswitha Merle, Uwe Roesler, Anika Friese

**Affiliations:** 1 Institute for Animal Hygiene and Environmental Health, Freie Universität Berlin, Berlin, Germany; 2 Department of Biological Safety, German Federal Institute for Risk Assessment, Berlin, Germany; 3 Department Food Safety, Federal Office of Consumer Protection and Food Safety, Berlin, Germany; 4 Centre for International Health Protection, Robert Koch Institute, Berlin, Germany; 5 Institute for Veterinary Epidemiology and Biostatistics, Freie Universität Berlin, Berlin, Germany

**Keywords:** ESBL, pAmpC, *Escherichia coli*, Broiler chicken, Cleaning and disinfection, Intervention, Whole genome sequencing, Transmission

## Abstract

**Background:**

Broiler chickens are frequently colonized with Extended-Spectrum Beta-Lactamase- (ESBL-) and plasmid mediated AmpC Beta-Lactamase- (pAmpC-) producing Enterobacterales, and we are confronted with the potential spread of these resistant bacteria in the food chain, in the environment, and to humans. Research focused on identifying of transmission routes and investigating potential intervention measures against ESBL- and pAmpC- producing bacteria in the broiler production chain. However, few data are available on the effects of cleaning and disinfection (C&D) procedures in broiler stables on ESBL- and pAmpC- producing bacteria.

**Results:**

We systematically investigated five broiler stables before and after C&D and identified potential ESBL- and pAmpC- colonization sites after C&D in the broiler stables, including the anteroom and the nearby surrounding environment of the broiler stables. Phenotypically resistant *E. coli* isolates grown on MacConkey agar with cefotaxime were further analyzed for their beta-lactam resistance genes and phylogenetic groups, as well as the relation of isolates from the investigated stables before and after C&D by whole genome sequencing. Survival of ESBL- and pAmpC- producing *E. coli* is highly likely at sites where C&D was not performed or where insufficient cleaning was performed prior to disinfection. For the first time, we showed highly related ESBL-/pAmpC- producing *E. coli* isolates detected before and after C&D in four of five broiler stables examined with cgMLST. Survival of resistant isolates in investigated broiler stables as well as transmission of resistant isolates from broiler stables to the anteroom and surrounding environment and between broiler farms was shown. In addition, enterococci (frequently utilized to detect fecal contamination and for C&D control) can be used as an indicator bacterium for the detection of ESBL-/pAmpC- *E. coli* after C&D.

**Conclusion:**

We conclude that C&D can reduce ESBL-/pAmpC- producing *E. coli* in conventional broiler stables, but complete ESBL- and pAmpC- elimination does not seem to be possible in practice as several factors influence the C&D outcome (e.g. broiler stable condition, ESBL-/pAmpC- status prior to C&D, C&D procedures used, and biosecurity measures on the farm). A multifactorial approach, combining various hygiene- and management measures, is needed to reduce ESBL-/pAmpC- *E. coli* in broiler farms.

**Supplementary Information:**

The online version contains supplementary material available at 10.1186/s12866-024-03292-7.

## Background

Colonization of broiler chickens with Extended-Spectrum Beta-Lactamase- (ESBL-) and plasmid mediated AmpC Beta-Lactamase- (pAmpC-) producing Enterobacterales is frequently detected throughout the livestock production chain [1–7]. Since day-old broiler chickens are already colonized with these resistant bacteria and high prevalence are frequently detected in broiler chicken farms, transmission routes were thoroughly investigated [8]. Horizontal and vertical transmission routes in the hatchery, from the environment to the farm, and from farm to farm have been described, illustrating the diversity of possible introduction routes into broiler chicken farms [9]. In addition to the different colonization routes, it was shown that even 10^1^ − 10^2^ colony forming units (cfu) of ESBL- and pAmpC- producing *Escherichia coli* (*E. coli*) are sufficient for colonization of day-old broiler chickens [10, 11]. In broiler farms, ESBL- and pAmpC- prevalence in newly hatched day-old broiler chickens appears to be low, and prevalence often increases throughout the fattening period [12]. Therefore, intervention measures are being investigated to prevent initial colonization or reduce the prevalence of ESBL- and pAmpC- producing bacteria in broiler chickens. Various interventions affecting broiler management or broiler gut composition have been studied with variable results on broiler chicken colonization rates [13–17]. Other important interventions include biosecurity measures that target introduction (external biosecurity) or spread within the farm and between flocks (internal biosecurity) [18, 19]. Here, cleaning and disinfection (C&D) procedures are of utmost importance to reduce the load of ESBL- and pAmpC- producing bacteria, but few data are available for broiler farms. Inadequate C&D after a fattening period can result in survival of ESBL- and pAmpC- producing bacteria in the broiler stable. Even small amounts of these resistant bacteria that are accessible to broiler chickens can lead to colonization of newly housed day-old broiler chickens. We therefore investigated the hazard of cleaned and disinfected broiler stables serving as a reservoir for ESBL- and pAmpC- producing bacteria. Our aim was to identify locations of ESBL- and pAmpC- survival within broiler stables and their nearby environment having the potential to subsequently colonize newly housed day-old broiler chickens.

## Materials and methods

### Sampled stables

Conventional broiler stables of identical construction belonging to the same company were screened for ESBL-/pAmpC- producing *E. coli* at the end of a broiler fattening period (day 23 to 31 of production). Fifty stables were screened and out of 21 ESBL-/pAmpC- positive broiler stables, five were selected on the basis of detected ESBL-/pAmpC- genes (see ‘Laboratory methods’) and information about the service period (e.g. duration of service period and disinfectants used, Table 1) to evaluate the efficacy of C&D procedures to eliminate ESBL-/pAmpC- producing *E. coli* from these stables. Sampling was conducted between April and October 2016. The sampled stables belonged to three different farms located within two kilometers of each other. Stable A and stable D belonged to one farm as well as stable B and stable C. Stable E was located on a third farm. On each farm, stables were freestanding with a minimum distance of 20 m from other stables, and two roads were used to transport goods to the stables (road A; feed, litter, hatchlings) or from the stables (road B; manure, fattened broiler chickens). Each broiler stable had an effective area of 1000 m^2^ with an attached anteroom that served as a hygiene lock and for storage of stable-specific equipment. Both the inner stables and the anterooms were made of concrete, having inside riveted steel plates on the ceiling and the walls (above a level of 50 cm from the floor).

**Table 1:** Disinfection procedures of the five investigated broiler stables

Stable	Sampling date	Farm	Service period	1st disinfection		2nd disinfection	
				Disinfectant	Concentration	Disinfectant	Concentration
A	04/2016	1	12 d	Formaldehyde	7.4%	Aldekol Des® 03	2%
B	06/2016	3	6 d	Formaldehyde	7.4%	Calcium hydroxide	24%
C	07/2016	3	6 d	Formaldehyde	7.4%	Aldekol Des® 03	2%
D	09/2016	1	8 d	Formaldehyde	7.4%	Aldekol Des® 03	2%
E	10/2016	2	10 d	Formaldehyde	7.4%	Calcium hydroxide	24%

Investigated stables A - E were located on three different farms. The duration of cleaning and disinfection service period is shown in days (d) together with the applied disinfectants and final concentration in percent (%)

### Cleaning and disinfection procedure

C&D of broiler stables was performed between two fattening periods by staff members who did not change throughout the study, each person being responsible for one step. The applied C&D corresponds to the commonly applied procedure for C&D in broiler stables and was performed within 6 to 12 days in four steps: (i) dry cleaning, (ii) wet cleaning, (iii) first disinfection, and (iv) second disinfection. Dry cleaning was performed with a front-end loader after housing-out of broiler chickens. A high-pressure washer with cold water and an all-purpose detergent (Grundreiniger, Weber-Chemie GmbH, Gladbeck, Germany) was used for wet cleaning and carried out by a single person. After a minimum drying time of at least 12 h after wet cleaning, the first disinfection was carried out with a fogging device using formaldehyde (Formaldehydlösung BIOZID PA 3, methanolarm stab., Chemie-Vertrieb GmbH & Co. KG, Hannover, Germany) and an exposure time of 4 h at > 28 °C. The second disinfection was performed 24 h after the first disinfection with two different disinfectants used for the five investigated houses (Aldekol DES® 03 or calcium hydroxide, Table 1). The disinfectants were selected by the broiler producer and represent agents commonly applied in livestock farming. We sampled each empty broiler stable 24 h after the second disinfection, right before preparations for the subsequent broiler flock.

### Samplings of the stables

**Fig. 1:**
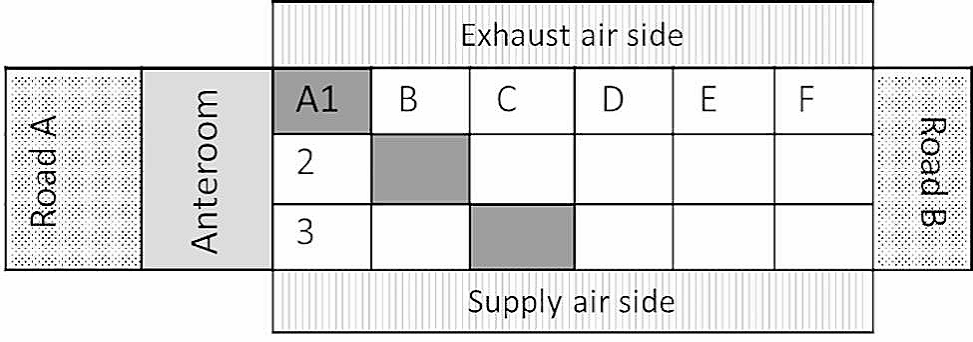
Schematic top view of investigated broiler stables. Stables were structured in length (A-F) and width (1–3) into 18 sections for a reproducible sampling scheme after cleaning and disinfection. The sampling location “A1, B2, C3” is highlighted in dark grey as an example. Together with anteroom and equipment, each of the five investigated broiler stables was sampled following the sampling scheme. Boot swabs outside the broiler stable were taken from supply airside and exhaust airside as well as from road A (transport of goods to stable) and road B (transport of goods off stable)

Each stable was sampled twice. The first samples were taken close to the end of the fattening period to assess the ESBL-/pAmpC- status of the current broiler flock using boot swab (VWR, Darmstadt, Germany; equal to ‘boot socks’) and pooled feces samples. The boot swab was taken by walking up and down the entire stable, while ten fresh droppings were collected along the entire length of the barn for the pooled feces sample. A stable was considered positive if ESBL- or pAmpC- producing *E. coli* were detected in the boot swab and/or the pooled feces sample. The second sampling was performed after C&D of the ESBL-/pAmpC- positive broiler stable and before preparations for the subsequent broiler flock (dry and empty stable without litter and feed). Stables’ inside (where broiler chickens are kept), the attached anteroom, and the surrounding environment were sampled with twisted gauze swabs (30 × 30 cm; Henry Schein, Berlin, Germany), boot swabs, and rinse water was collected (Supplementary Table 1). Prior to each sampling, autoclaved gauze swabs or boot swabs were transferred to sterile bags (Carl Roth, Karlsruhe, Germany) and moistened under sterile conditions with 5 ml or 10 ml phosphate buffered saline (PBS; Phosphate Buffered Saline tablets, Thermo Fisher Diagnostics GmbH, Wesel, Germany). No neutralizer for inactivation of disinfectant residues was added. Depending on the location, single gauze swabs or two to three pooled gauze swabs were used for one sample, and each gauze swab was used to sample a 10 × 10 cm area. Thirty-seven different sampling locations inside the stable, 21 different sampling locations in the anteroom and four different sampling locations in the surrounding environment were sampled to investigate various locations from ceiling to floor in each stable (Supplementary Table 1). To ensure comparability of samples between the five stables investigated, a precise sampling scheme was used that systematically structured the stable into sections (A - F and 1–3; Fig. 1, Supplementary Table 1). In addition, boot swabs were taken from stable’s floor, anteroom’s floor and from each of the four surrounding sides of the broiler stable (by walking up and down both sides of stable’s and anteroom’s floor and walking the entire length of each surrounding side), and one rinse water sample (75 ml) was collected from the anterooms’ floor drain using a sterile syringe. In total, the 69 samples reflect all the different materials (e.g. concrete, metal, plastic and organic origin), objects (e.g. removable and non-removable) and locations (e.g. floor, wall, ceiling of stable and anteroom) in and around a stable to systematically investigate the risk of ESBL-/pAmpC- detection in broiler farms (Supplementary Table 1).

### Laboratory methods

*Sample preparation*. On the collection day, each gauze swab or boot swab was mixed with 20 ml or 200 ml Luria Bertani broth (LB; Carl Roth, Karlsruhe, Germany), and 3 ml of rinse water or 20 g of pooled feces were mixed in a ratio of 1:10 with LB before homogenizing for 2 min at 200 rounds per minute in a stomacher (except rinse water). Enrichment was performed 18–24 h at 37 °C and 10 µl were streaked out on MacConkey agar no. 3 (Fisher Scientific GmbH, Schwerte, Germany) containing 1 µg/ml cefotaxime (AppliChem, Darmstadt, Germany) and on agar plates without antibiotic supplementation. Agar plates were incubated 24 h at 37 °C for qualitative analysis of ESBL- and pAmpC- producing and non-resistant *E. coli*. Two phenotypically suspected ESBL- and pAmpC- producing *E. coli* isolates per colony morphology and sample were stored at -80 °C for further analyses. Additionally, samples taken after C&D were quantitatively and qualitatively analyzed on bile aesculin azide agar (BAA; Fisher Scientific GmbH, Schwerte, Germany) to detect residual enterococci. Enrichment was performed as described above and streaked out on BAA. For enterococci quantification, non-enriched gauze swab and boot swab samples were 1:10 diluted in PBS, and appropriate dilutions were plated on BAA and 50 ml of rinse water was filtered in a bottle top filter (Nalgene, Fisher Scientific GmbH, Schwerte, Germany) using 0.22 μm nitrocellulose filter (Carl Roth, Karlsruhe, Germany) which was then transferred to BAA agar. BAA agar plates were incubated for 48 h at 37 °C and two dilution levels were counted for quantification.

*Species identification*. Isolates’ species was identified using matrix-assisted laser desorption time of flight (MALDI Microflex LT and Biotyper database; Bruker Daltonics, Bremen, Gemany).

*Phylogenetic grouping and determination of ESBL- and pAmpC- genes*. Multiplex PCR was performed to determine the phylogenetic group [20] with modifications according to Projahn et al. [5]. To identify ESBL-/pAmpC- genes, real-time PCR was performed [21] and identified ESBL-/pAmpC- genes (*bla*_TEM_, *bla*_SHV_, *bla*_CTX−M_, *bla*_CMY_) were verified by sequencing of at least one isolate per sample according to Projahn et al. [5]. DNA was extracted by boiling method.

*Pulsed-field gel electrophoresis*. 27 *E. coli* isolates taken before (first sampling) and after C&D (second sampling) of the five investigated broiler stables harboring identical ESBL-/pAmpC- genes and belonging to the same phylogenetic group were further analyzed by pulsed-field gel electrophoresis (PFGE) according to Projahn et al. [5]. XbaI-PFGE patterns were grouped by presence or absence of more than three bands, resulting in a new PFGE-number (I-X). Minor differences in one to three bands resulted in a new subgroup labelled by the letters a-d (Supplementary Fig. 1).

*Whole genome sequencing (WGS) and bioinformatic analyses.* 21 *E. coli* isolates taken before (first sampling) and after C&D (second sampling) from stables B – E harboring the same resistance gene and phylogenetic group which clustered together in PFGE were whole genome sequenced to further investigate their phylogenetic relationship (six isolates did not cluster together in PFGE and were excluded). No samples from stable A were whole genome sequenced as they did not cluster together with any other isolate in PFGE. DNA was extracted with Invitrogen PureLink Genomic DNA Mini Kit (Thermo Fisher Scientific, Wesel, Germany) and WGS was performed by Illumina NextSeq 300-bp paired-end with a coverage between 80x and 100x (LGC Genomics GmbH, Berlin, Germany). Raw reads were uploaded to EnteroBase for data pre-processing and de novo assembly. Genome sequence data of *E. coli* isolates are publicly available in the NCBI Sequence Read Archive under the BioProject Accession number PRJNA1044304 (http://www.ncbi.nlm.nih.gov/bioproject/1044304). Phylogenetic analyses of *E. coli* strains were performed using single nucleotide polymorphism (SNP) and core-genome multi-locus sequence typing (cgMLST) based on 2513 loci using EnteroBase and visualized with GrapeTree and MS Tree V2 algorithm [22, 23]. MLST sequence types and phylogroups were assigned using EnteroBase [24, 25]. Serotypes were predicted with EnteroBase and ambiguous results were checked against the EcOH database [26]. Antibiotic resistance determination was done with the *E. coli* functional genotyping tool (version 1.2) implemented in BioNumerics 7.6.3 (Applied Math, Sint-Martens-Latem, Belgium).

### Statistical analyses

Statistical analyses were performed using SPSS Statistics 25 (IBM, New York, USA). All 69 different sampling locations from inside of the stable, anteroom and surrounding environment were grouped to 14 categories to analyze the detection frequencies of ESBL-/pAmpC- *E. coli* and quantities of enterococci (‘*stable floor’, ‘stable wall’, ‘stable ceiling’, ‘stable interior’, ‘stable ventilation system’, ‘anteroom floor’, ‘anteroom wall’, ‘anteroom ceiling’, ‘anteroom interior’, ‘anteroom rinse water’, ‘anteroom door’, ‘surrounding floor’, ‘surrounding tractor tires’, ‘surrounding ventilation system’*; Supplementary Table 1). The grouping was based on the three different areas ‘stable’, ‘anteroom’ and ‘surrounding environment’, which have different requirements for C&D (i.e. no C&D of the surrounding environment and gentle C&D of the anteroom due to electrical devices and standard C&D of the stable) resulting in a different probability of ESBL-/pAmpC- detection. Within these three areas, grouping was based on locations (e.g. floor, wall, ceiling) as it was expected that sampling locations near the floor have a higher probability of ESBL-/pAmpC- detection due to fecal contamination. Quantitative data of enterococci were log10 transformed and grouped into the categories no detection or low detection rate (detection in enrichment to < 10^7^ cfu/swab) of enterococci and high detection rate (≥ 10^7^ cfu/swab) of enterococci for statistical analyses. Supplementary Table 3 gives an overview of enterococci negative sampling locations, detection via enrichment and swabs with enterococci quantities of < 10^7^ cfu/swab and ≥ 10^7^ cfu/swab.

Chi-square test and Fisher’s exact test (if 25% or more of the cells have expected values below 5) were used to compare the detection frequencies of ESBL- and pAmpC- producing *E. coli* and enterococci inside the stable, anteroom and environment as well as between the defined categories. Concerning the comparison between ESBL-/pAmpC- *E. coli* (present or not) and enterococci (no or low detection (< 10^7^ cfu/swab) vs. high detection rate (≥ 10^7^ cfu/swab)), odds ratios including 95% confidence intervals (CI) were calculated.

The significance level was set to 0.05.

## Results

ESBL- and pAmpC- producing *E. coli* were detected in four out of five investigated broiler stables after C&D (stables B, C, D and E). Each stable showed highly related isolates taken before and after C&D using cgMLST. One stable positive for ESBL-producing *E. coli* before C&D was tested negative after C&D (stable A). Detected ESBL- and pAmpC- resistance genes in the four positive broiler stables were *bla*_CTX−M−1_ (stables C, D and E) and *bla*_CMY−2_ (stable B) with phylogenetic groups A/C (stable B), E/D (stable B) and F (stable B, C, D and E) detected in PCR (Supplementary Table 2). Using WGS, further resistance determinants of the investigated *E. coli* isolates from stable B, C and E were identified (Supplementary Table 2). Isolates belonging to one stable (stable B, C or E) harbored identical resistance genes, most likely due to acquisition of resistance plasmids. Interestingly from stable C, only three of the six isolates harbored additional resistance determinants. The remaining three isolates from stable C (one before C&D, two after C&D: tractor tires and road B) and all isolates from stable D only carried the ESBL resistance gene. Additionally, WGS revealed the phylogenetic groups A (stable B) and G (stable B, C, D and E) and serotypes O68:12 (stable B), O132:H4 (stable B), Onovel12:H4 (stable C and D) and O143:H4 (stable E) (Supplementary Table 2).

After C&D the ESBL-/pAmpC *E. coli* detection rate of all investigated samples was 4.9% (*n* = 17 out of 345). Highest ESBL-/pAmpC- *E. coli* rate was detected in the surrounding environment of the broiler stables (20%, *n* = 8 out of 40) which was significantly higher compared to the stable (3.7%, *n* = 7 out of 190) and the anteroom (1.7%, *n* = 2 out of 115) (*p* < 0.001, Fisher’s exact test, Supplementary Table 1). The ESBL-/pAmpC- detection rate was highest in the categories *‘stable floor’* (20% detection rate, *n* = 7 out of 35; floor cracks, transition between floor and wall, wooden board at entry) and *‘surrounding floor’* (35% detection rate, *n* = 7 out of 20; boot swabs), with *‘stable floor’* positive in four out of five tested stables and *‘surrounding floor’* positive in three out of five tested stables. Further ESBL-/pAmpC- positive categories were *‘anteroom floor’* (floor cracks), *‘surrounding tractor tires’* (tractor used for litter supply) and *‘anteroom interior’* (sink’s drain). All other categories were negative for ESBL-/pAmpC- *E. coli* in the five investigated broiler stables after C&D. Noteworthy, from ESBL-/pAmpC- negative sampling locations of the investigated broiler stables, a proportion of 10.0% (*n* = 34 out of 345) of samples showed growth of *E. coli* on non-selective agar plates after C&D, indicating a survival of *E. coli* on these spots (stable floor, cable lines, nipple drinkers, feed trough, anteroom floor, anteroom door, sink’s drain, rubber boots, dismantled metal boxes from stable, trash bin lid, ventilation flap, tractor tires and surrounding floor; Supplementary Table 1). The overall detection rate of (non-selectively grown) *E. coli* in all investigated samples, including ESBL-/pAmpC- positive samples, was 14.8% (*n* = 51 out of 345; with 47.5% (*n* = 19 out of 40) of samples from the surrounding environment, 11.1% (*n* = 21 out of 190) from stable, 9.6% (*n* = 11 out of 115) from anteroom). Although this study is focused on ESBL-/ pAmpC- *E. coli* it is worth mentioning that we detected one TEM-52 producing *Klebsiella pneumoniae* on farm C what implies that multiple ESBL-resistance genes can be present on one broiler farm at the same time (data not shown).

**Fig. 2:**
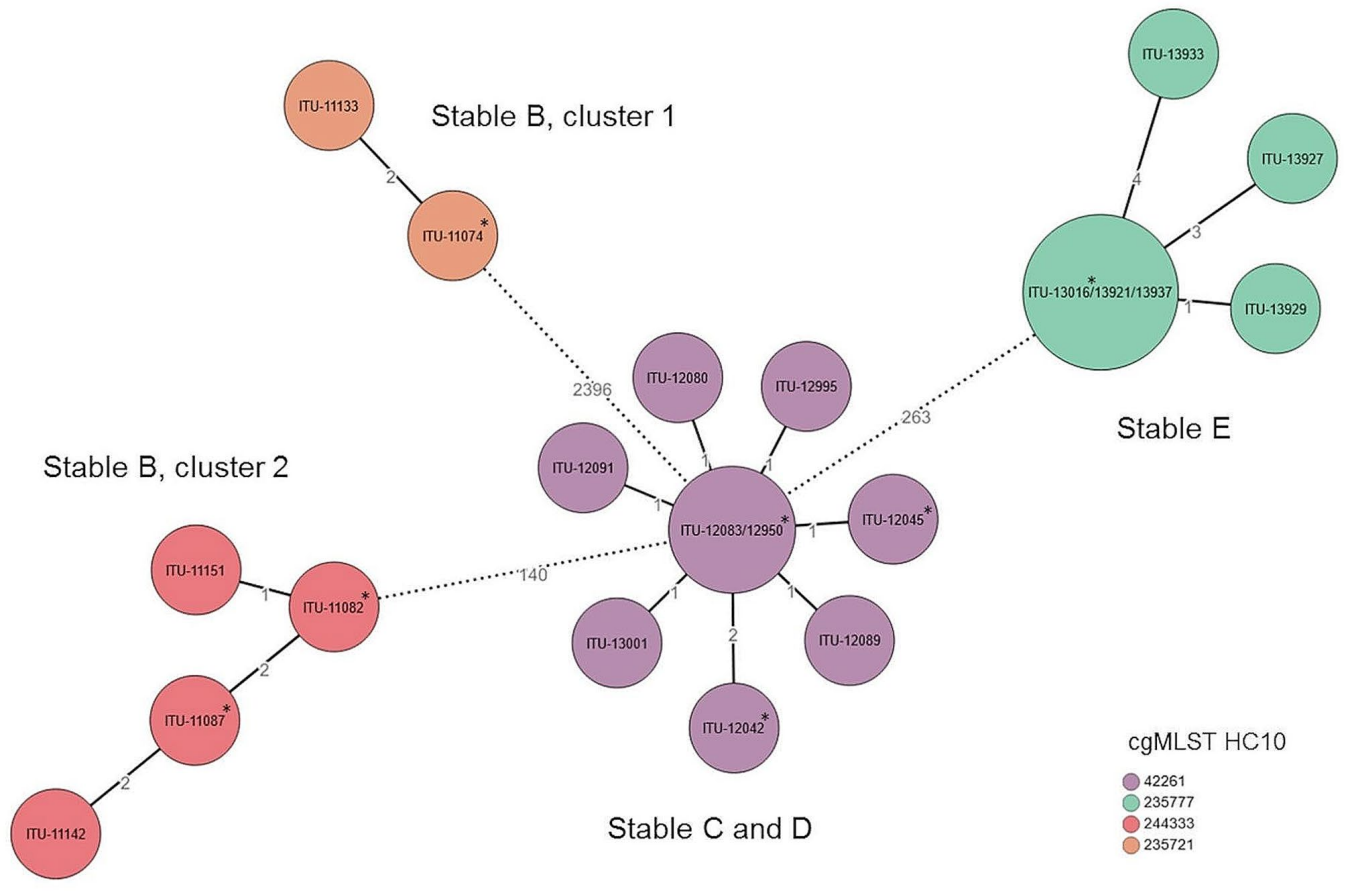
Core genome multilocus sequence typing (cgMLST) of ESBL- and pAmpC- producing *E. coli* isolates. Isolates are from four investigated broiler stables B – E before and after cleaning and disinfection which clustered together in PFGE. Isolates are colored according to the cgMLST hierarchical clustering (HC) 10, indicating that the four clusters include all strains with links no more than 10 alleles difference. Information on each sample is given in Supplementary Table 2. * Isolates detected before cleaning and disinfection of broiler stables

cgMLST revealed highly related isolates taken before and after C&D for stables B, C, D and E differing in less than 5 alleles by hierarchical clustering between related isolates for stables B, C and D. For stable E, isolates clustered with less than 10 allele’s difference in cgMLST (Hierarchical Cluster (HC) 10; Fig. 2). SNP analyses for isolates of each cgMLST cluster confirmed phylogenetic relationships (Supplementary Fig. 2). In stable B, two distinct cgMLST clusters of pAmpC- *E. coli* were detected, differing in at least 2381 alleles, with both clusters harboring isolates from before and after C&D. The pAmpC- *E. coli* after disinfection of stable B were detected in the stable (cluster 1, transition floor/wall) and the close environment of the broiler stable (cluster 2, boot swabs). Samples from stable C harbored highly related isolates after C&D from inside the stable (transition floor/wall), anteroom (sink’s drain) and the surrounding environment (tractor tires and boot swab). All isolates of stable C clustered together with all isolates of stable D in the cgMLST analysis. SNP analysis of these isolates revealed no distinct separation of the stable C and stable D isolates indicating transmission events between the two stables (Supplementary Fig. 2). As the stables belong to different broiler farms and ESBL- positive samples were only detected inside stable D (transition floor/wall and floor cracks), a transmission from farm (stable C) to farm (stable D) via shared equipment or the presence of a common ESBL- source (e.g. tractor) is highly likely. The described transmission event is also supported by the additional resistance determinants of the isolates from stable C and stable D (see above), as the resistance profile of the tractor tires and road B isolates from stable C is identical to the profile of stable D isolates. Finally isolates from stable E clustered together with isolates from inside stable (transition floor/wall and floor cracks) and the close environment of the broiler stable (boot swabs).

The qualitative analysis of enterococci revealed significant differences between the locations with higher enterococci detection rates in the surrounding environment and lower detection rates in the anteroom (*p* < 0.001, chi-squared test, Supplementary Table 3). Comparing the enterococci quantities of the different categories, the highest detection rates were found in *‘stable floor’*, *‘surrounding floor’* and *‘surrounding tractor tires’* (*p* < 0.001, chi-squared test), with more frequent detection of high enterococci quantities (10^7^ cfu/ swab) in these categories. The probability to detect ESBL-/ pAmpC- *E. coli* was 73.7 times higher in samples where high enterococci quantities were detected compared to those where no or low quantities were detected (*p* < 0.001, chi-squared test; 95% CI 9.6–566.5).

## Discussion

The applied C&D procedures did not eliminate ESBL-/pAmpC- producing *E. coli* from the broiler stables studied, and highly related isolates were detected before and after C&D. Only one of five positive broiler stables before C&D was negative for ESBL-/pAmpC- producing *E. coli* after C&D (stable A). This result is in accordance with other studies in which elimination of *E. coli* was not achieved after C&D procedures [27–29]. Each of the four ESBL- and pAmpC- positive broiler stables after C&D had highly related resistant *E. coli* isolates before and after C&D according to cgMLST, emphasizing survival and/or transmission of bacteria on broiler farms (stable B - E). Carryover of resistant bacteria from positive broiler stables to the anteroom and surrounding environment or vice versa is possible. Especially when broilers are housed and the bacterial load in the stable is high or when broilers are housed-out and C&D procedures start (e.g. dry cleaning), carryover from the stable is possible as broiler chicken flocks still regularly tested positive for ESBL-/pAmpC- producing *E. coli* [30–32], as do environmental samples from the adjacent area of broiler stables, which can lead to the introduction of bacteria into the stable at any time. Environmental samples carrying ESBL-/pAmpC- *E. coli* include barn equipment, dust, air, rinse water, surface water, and soil [8, 12, 33–36]. We identified cracks in the stable’s floor, the transition between the stable’s floor and wall, the sink’s drain in the anteroom, and tractor tires used for litter supply, as well as the broiler stable’s surrounding floor as sources of ESBL-/ pAmpC- producing bacteria. The ESBL-/pAmpC- positive spots indicate that the floor and equipment in contact with the floor require special attention during C&D procedures. One possible approach is to repair defects in the stable’s floor and a thorough C&D of the equipment and surrounding floor whenever possible (e.g. roads for the transportation of goods). It was already shown that poor floor quality (e.g. cracks in concrete floor) is a significant risk factor for broilers’ mortality, as these spots cannot be properly cleaned and disinfected, resulting in the survival of pathogens that can then cause disease in subsequent flocks [37]. Since the required colonization dose for ESBL-/ pAmpC- producing bacteria in day-old broiler chickens is as low as 10^1^ − 10^2^ cfu, the likelihood of ESBL-/pAmpC- colonization of newly housed broiler flocks originating from such sites is very high [10]. A major reason for bacterial survival is contamination of stables with organic matter that has not been removed by cleaning procedures prior to disinfection, as organic matter reduces the effectiveness of disinfectants, resulting in bacterial survival [27, 38]. In agreement, non-selectively grown *E. coli* was mostly detected on spots with contact to the floor or close to the floor. A possible explanation for the detection of *E. coli* on ventilation flaps, trash can lids, and cable lines may be settled dust that was not removed by C&D, as *E. coli* can survive in dust for several years [39]. In general, we have to consider that each farm or integrated production has different biosecurity measures, different cleaning protocols with personnel performing C&D or external companies contracted for different work [40]. The C&D in this study was performed under field conditions and reflects the common procedure in broiler farming. The cleaning agents and disinfectants used were suitable for preventive measures, whereby Aldekol Des® 03 was only used at temperatures above 10 °C (not effective at lower temperatures) [41]. For all biosecurity measures, including C&D, compliance by staff members is of utmost importance to achieve satisfactory results. Once a biosecurity concept has been developed, it must be communicated to all employees and consistently monitored for compliance and improvement. As we detected highly related isolates in distinct stables (stable C and D), an improvement of the biosecurity measures seems possible. In addition, each broiler stable has different structural preconditions, such as the age of the buildings and the construction material, with all of the above factors affecting the initial bacterial contamination of the broiler stable and the effectiveness of C&D procedures [27, 42].

Isolates before and after C&D from stables B - E clustered together with less than 10 alleles difference in cgMLST, which displays short-term, single source transmissions of *E. coli* [43]. In stable B, two cgMLST clusters of pAmpC- *E. coli* were detected, both harboring isolates from before and after C&D. The results from stable B demonstrate that distinct ESBL-/pAmpC- *E. coli* strains can be detected together in a broiler stable [12] and that ESBL-/pAmpC- *E. coli* can survive C&D procedures within the stable (stable B, cgMLST cluster 1, HC10, Fig. 2) or be disseminated to the close surrounding environment of the stable or introduced into the stable from the close surrounding environment (stable B, cgMLST cluster 2, HC10, Fig. 2). Similarly, in stables D and E, survival of ESBL- *E. coli* within the stable and dissemination from or introduction into the stable was detected. Horizontal transmission routes at farm level were described extensively, but survival of ESBL-/pAmpC- *E. coli* after C&D of broiler stables with detection of highly related isolates in and around the stables was not shown previously [9, 12, 44, 45]. The high relation of isolates from stables C and D with two indistinguishable isolates using cgMLST and the fact that all samples from stables C and D cluster together in cgMLST and differ by less than 5 alleles underscores the risk of transmission of resistant *E. coli* between distinct broiler farms or the presence of a common source leading to ESBL- detection in both stable C and D. Another important factor in resistance transmission is plasmid-mediated resistance. Plasmids play a major role in *E. coli* with dissemination of these mobile genetic elements from resistant strains to other bacteria, as well as in plasmid acquisition [46]. In our study, isolates from three of four ESBL-/pAmpC- positive broiler stables harbored additional resistance determinants after C&D, most likely due to plasmid acquisition. The complex interactions of resistance gene transfer are well seen in isolates of stable C, as only three of six investigated isolates harbored additional resistance determinants. Since plasmid acquisition is not depicted in cgMLST or SNP analyses, isolates of stables C and D with distinct resistance profiles are undistinguishable in these analyses. Biosecurity measures were implemented in all broiler farms studied. Measures included restricted access of vehicles and visitors to the farm, with a hygiene lock to be passed before entering. Each broiler stable had a shoe disinfection bath, sink, stable-specific equipment (except heavy machines), and rodent control was applied. However, strict compliance to biosecurity measures is required [47]. One possible route of transmission from stable C to stable D or the presence of a common source is the contaminated tractor tires that tested positive for ESBL- *E. coli* in stable C after C&D procedures, as some equipment such as heavy machines was shared between the farms. Further biosecurity measures such as cleaning and disinfection baths for shared heavy machines before entering distinct farms could minimize the risk of inter-farm transmission if exposure times are met.

To date, only one study investigated the presence of ESBL-producing *E. coli* after C&D procedures in broiler stables, but did not investigate transmission routes during C&D procedures [29]. The study by Benameur et al. (2023) detected ESBL-producing *E. coli* in 3.8% of 104 stables investigated using 104 enriched swab samples after C&D, but did not use selective laboratory methods to detect ESBL-/pAmpC- producing *E. coli* because they investigated the presence of multidrug-resistant *E. coli* in cleaned and disinfected poultry stables. The overall detection rate of *E. coli* after C&D was very high in the study by Benameur et al. with 88.46%. One reason for the high detection rate could be the long vacancy period of three to six weeks between C&D and the sampling, which makes (re)contamination likely. We used moistened gauze and boot swabs to sample the cleaned and disinfected stables and selectively enriched the samples overnight because we expected low growth of ESBL-/pAmpC- *E. coli* immediately after C&D procedures. Moistened gauze swabs and enrichment can lower the detection limit compared to contact agar plates, which are regularly used to evaluate C&D procedures [28]. Another approach to lower the detection limit is the addition of neutralizers to sampling materials and agar plates, as these inactivate disinfectant residues. As expected, only 4.9% of all samples tested were positive for ESBL-/pAmpC- *E. coli* after enrichment, and the positive sampling locations were difficult to access. In a study by Luyckx et al. [38], C&D procedures reduced the *E. coli* detection rate of enriched samples in broiler stables from 93% before cleaning to 7% after disinfection. Since high *E. coli* detection rates are expected before C&D due to fecal contamination, the detection rate should decrease significantly due to the applied C&D measures. We did not investigate the quantitative occurrence of ESBL-/pAmpC- *E. coli* before C&D as we aimed to trace possible transmission routes from positive broiler flocks during C&D procedures. In our study, the ESBL-/pAmpC- detection rate after C&D in all samples tested was 4.9% with a total *E. coli* detection rate of 14.8%. The high detection rate is caused by the samples from the surrounding environment (20% ESBL-/pAmpC- *E. coli*; 47.5% total *E. coli*), as C&D procedures are only applied in the stable and anteroom and not in the surrounding environment. It is worth mentioning that we only included initially ESBL-/ pAmpC- positive broiler stables in our study, which might increase the detection rate after C&D compared to initially negative broiler stables [40].

We showed a significantly higher detection probability of ESBL-/pAmpC- *E. coli* on sampling locations with high enterococci quantities. In our analysis, due to power restrictions we did not consider that samples from different locations in and around a stable and farm might be related to each other, but we assume that the effect would be statistically significant even in a random factors model. *Enterococcus* spp. is commonly used as an indicator bacterium for fecal contamination and has been shown to detect organic matter after C&D procedures [28, 48]. Samples from the floor and from the immediate environment of broiler chickens, where organic matter can accumulate (e.g. cracks and crevices in the floor), showed higher enterococci quantities in our study (Supplementary Table 3). These spots contribute to residual contamination and biofilm formation and require spot treatment or double-strength disinfection for effective C&D [27]. It was shown that the effectiveness of disinfectants against biofilms is lower compared to planktonic cells and a longer exposure time of the disinfectants is recommended [49]. As the effectiveness of commercially available disinfectants is usually only tested on planktonic cells, the test methods must be adapted to practical scenarios and biofilms must also be examined. Enterococci quantities were also high on spots that were not regularly cleaned and disinfected (e.g. wooden board at the stable’s entrance, sink’s drain in the anteroom, tractor tires used for litter supply, and the broiler stables’ surrounding environment). C&D of these spots may be too laborious or there may be no knowledge of the need for C&D procedures at these spots, resulting in bacterial survival. Therefore, detection of high *Enterococcus* spp. quantities at cleaned and disinfected spots in broiler stables is eligible to estimate the ESBL- and pAmpC- *E. coli* presence at these spots.

## Conclusion

Showing the survival of ESBL- and pAmpC- producing *E. coli* after C&D, we conclude that complete elimination of these resistant bacteria is unlikely in conventional broiler stables. Strict biosecurity measures and the implementation of improved disinfection procedures could enhance C&D results, but it seems likely that locations which are difficult of access remain positive. Prophylactic C&D procedures do not aim to “sterilize” broiler stables, but to reduce overall infection pressure and control infectious agents in livestock farming [28, 50]. C&D should be considered as one tool that, together with other hygiene and management measures, can reduce the presence of resistant bacteria in broiler farms.

### Electronic supplementary material

Below is the link to the electronic supplementary material.

Supplementary Material 1

Supplementary Material 2

Supplementary Material 3

Supplementary Material 4

## Data Availability

All data supporting this article are included within the article and the provided supplementary files. Genome sequence data of *E. coli* isolates are publicly available in the NCBI Sequence Read Archive under the BioProject Accession number PRJNA1044304 (http://www.ncbi.nlm.nih.gov/bioproject/1044304).
