# Supplementary figures and images for: Survival of highly related ESBL- and pAmpC- producing Escherichia coli in broiler farms identified before and after cleaning and disinfection using cgMLST
Source: BMC Microbiol. 2024 Apr 25;24:143. doi: 10.1186/s12866-024-03292-7 (PMC11044539; doi:10.1186/s12866-024-03292-7)

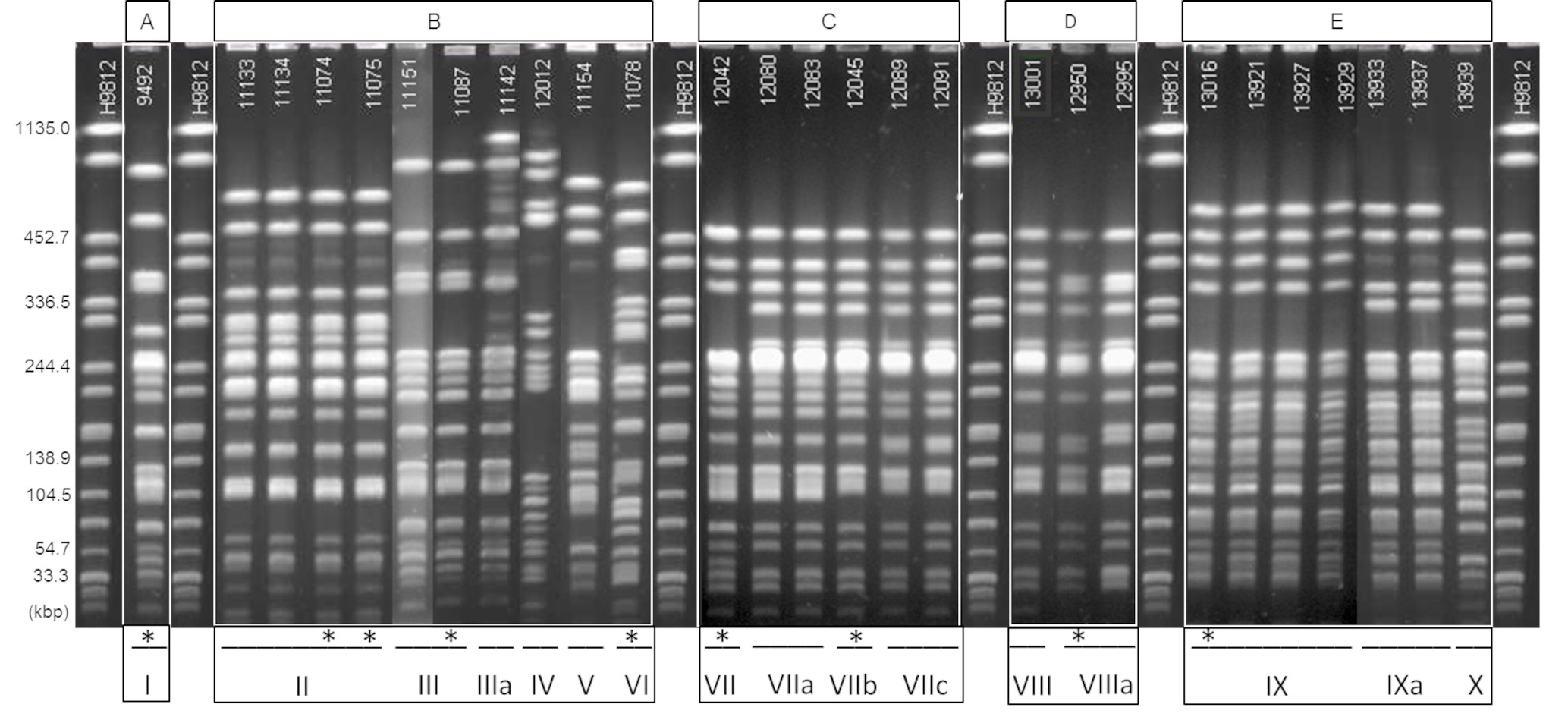

Supplement: Supplementary file 1 — Supplementary Material 1 [file 12866_2024_3292_MOESM1_ESM.jpg]

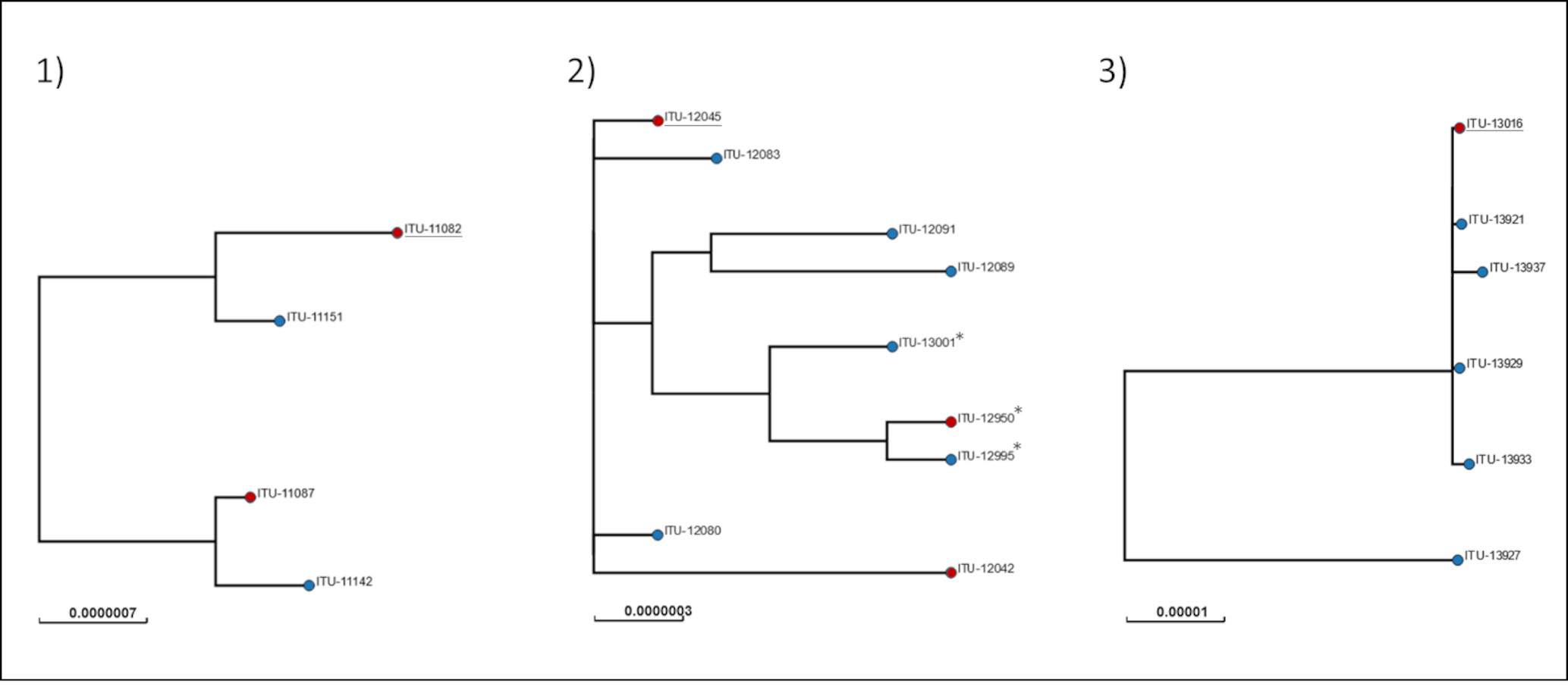

Supplement: Supplementary file 2 — Supplementary Material 2 [file 12866_2024_3292_MOESM2_ESM.jpg]
