# Supplementary material for: Survival of highly related ESBL- and pAmpC- producing Escherichia coli in broiler farms identified before and after cleaning and disinfection using cgMLST
Source: BMC Microbiol. 2024 Apr 25;24:143. doi: 10.1186/s12866-024-03292-7 (PMC11044539; doi:10.1186/s12866-024-03292-7)
Supplement: Supplementary file 3 — Supplementary Material 3 [file 12866_2024_3292_MOESM3_ESM.docx]

Supplementary information

Additional file 1: Supplementary Tables (.docx)

*Supplementary Table 1.* Detection rate of ESBL-/pAmpC- producing E. coli (bold print) and overall non-selectively detected E. coli (total E. coli) and enterococci (italic print) in the stable, anteroom and surrounding environment of the five investigated broiler stables (A-E) after cleaning and disinfection. *Supplementary Table 3*. Detection of *Enterococci* on sampling locations in five investigated broiler stables (A-E) after cleaning and disinfection based on grouping into 14 sampling categories.

*Supplementary Table 2.* Characterization of ESBL- and pAmpC- E. coli detected before and after cleaning and disinfection (C&D) of the five investigated broiler stables (A - E).

Additional file 2: Supplementary Figure 1 (.pdf)

*Supplementary Figure 1*. XbaI-PFGE pattern and clusters (I – X) of ESBL- and pAmpC- producing *E. coli* isolates from five investigated broiler stables A - E before (*) and after cleaning and disinfection harboring identical ESBL-/pAmpC- genes and belonging to the same phylogenetic group. H9812 = size standard *Salmonella enterica* serovar Braenderup H9812

Additional file 3: Supplementary Figure 2 (.pdf)

*Supplementary Figure 2*. SNP-based phylogenetic trees of ESBL-/pAmpC- E. coli from related isolates before (red circle) and after (blue circle) cleaning and disinfection using EnteroBase: (1) Isolates of stable B, cluster 2; (2) Isolates of stable C and D; (3) Isolates of stable E. Reference genomes are underlined. The tree is based on SNPs in non-repetitive regions present in 95 % of all genomes. The scale bar refers to the frequency of mutation per site in the core genome. SNP tree of stable B cluster 1 was not created, as a minimum of four sequences are required for SNP analysis. * Sequences of stable C
