# Supplementary material for: Survival of highly related ESBL- and pAmpC- producing Escherichia coli in broiler farms identified before and after cleaning and disinfection using cgMLST
Source: BMC Microbiol. 2024 Apr 25;24:143. doi: 10.1186/s12866-024-03292-7 (PMC11044539; doi:10.1186/s12866-024-03292-7)
Supplement: Supplementary file 4 — Supplementary Material 4 [file 12866_2024_3292_MOESM4_ESM.docx]

Supplementary Table 1: Detection rate of **ESBL-/pAmpC- producing E. coli** (**bold print**) and overall non-selectively detected E. coli (total E. coli) and enterococci (italic print) in the stable, anteroom and surrounding environment of the five investigated broiler stables (A-E) after cleaning and disinfection.

| **Location** | **Sample** | **Sampling category** | **Sampling location**  (Position in sampling scheme^1^) | **Stable** | | | | | **Average**  ***E. coli*** (%) | | **Average**  enterococci  (%) |
| --- | --- | --- | --- | --- | --- | --- | --- | --- | --- | --- | --- |
|  |  |  |  | **A** | **B** | **C** | **D** | **E** | |  |  |
| Stable | Boot swab  (n = 1) | Stable | Boot swab stable floor | - | - | - | - | - | | **20%**  40% | *68.6%* |
|  |  | floor |  |  |  |  |  |  |  |  |  |
|  | Gauze swab  (n = 37) | Stable | Floor drain (C2) | - | -^#^ | - | -^#^ | - | |  |  |
|  |  | floor | Floor cracks I (A1, B2, C3) | - | - | - | +*^#^ | -^#^ | |  |  |
|  |  |  | Floor cracks II (D1, E2, F3) | - | - | - | - | - | |  |  |
|  |  |  | Transition floor to wall I (A1, A3) | -^#^ | +°^#^ | -^#^ | -^#^ | -^#^ | |  |  |
|  |  |  | Transition floor wall II (F1, F3) | - | +°^#^ | +*^#^ | +*^#^ | +*^#^ | |  |  |
|  |  |  | Wooden board stable entry (A1) | - | +°^#^ | - | -^#^ | - | |  |  |
|  |  | Stable | Supply air side upper wall (A3, C3, E3) | - | - | - | - | - | | **0%**  0% | *34.3%* |
|  |  | wall | Supply air side wall medium height (A3, C3, E3) | - | - | - | - | - | |  |  |
|  |  |  | Supply air side lower wall (A3, C3, E3) | - | - | - | - | - | |  |  |
|  |  |  | Exhaust air side upper wall (A1, C1, E1) | - | - | - | - | - | |  |  |
|  |  |  | Exhaust air side medium wall (A1, C1, E1) | - | - | - | - | - | |  |  |
|  |  |  | Exhaust air side lower wall (A1, C1, E1) | - | - | - | - | - | |  |  |
|  |  |  | Door to stable, handle inside (A1) | - | - | - | - | - | |  |  |
|  |  | Stable | Ceiling I (A1, B2, C3) | - | - | - | - | - | | **0%**  0% | *33.3%* |
|  |  | ceiling | Ceiling II (D1, E2, F3) | - | - | - | - | - | |  |  |
|  |  |  | Ceiling corner I (A1, A3) | - | - | - | - | - | |  |  |
|  |  |  | Ceiling corner II (F1, F3) | - | - | - | - | - | |  |  |
|  |  |  | Ceiling above lights I (B1, B3) | - | - | - | - | - | |  |  |
|  |  |  | Ceiling above lights II (E1, E3) | - | - | - | - | - | |  |  |
|  |  | Stable | Inner feed pipe (A2) | - | - | - | - | - | | **0%**  5.3% | *42.7%* |
|  |  | interior | Heater (C1, D3) | - | - | - | - | - | |  |  |
|  |  |  | Cable lines (A1-3) | - | - | - | -^#^ | - | |  |  |
|  |  |  | Crank of water-/ feed pipes (A1, A3) | - | - | - | - | - | |  |  |
|  |  |  | Lights I (B1, B3) | - | - | - | - | - | |  |  |
|  |  |  | Lights II (E1, E3) | - | - | - | - | - | |  |  |
|  |  |  | Nipple drinkers I (A1, B2, C3) | - | - | -^#^ | - | -^#^ | |  |  |
|  |  |  | Nipple drinkers II (D1, E2, F3) | - | - | - | - | - | |  |  |
|  |  |  | Cord of drinking/feeding troughs I (A1, B2, C3) | - | - | - | - | - | |  |  |
|  |  |  | Cord of drinking/feeding troughs II (D1, E2, F3) | - | - | - | - | - | |  |  |
|  |  |  | Feed trough outside I (A1, B2, C3) | - | - | - | - | - | |  |  |
|  |  |  | Feed trough outside II (D1, E2, F3) | - | - | - | - | - | |  |  |
|  |  |  | Feed trough inside I (A1, B2, C3) | - | - | - | - | - | |  |  |
|  |  |  | Feed trough inside II (D1, E2, F3) | - | - | - | -^#^ | - | |  |  |
|  |  |  | Water tap (A3) | - | - | - | - | - | |  |  |
|  |  | Stable | Ventilator (A1, C1, E1) | - | - | - | - | - | | **0%**  13.3% | *60%* |
|  |  | ventilation | Metal box around ventilator (A1, C1, E1) | - | - | - | - | - | |  |  |
|  |  | system | Ventilation flap supply air (A3, C3, E3) | - | -^#^ | - | - | -^#^ | |  |  |
|  | **Sum** | **ESBL-/pAmpC- *E. coli*** | | **0%** | **7.9%** | **2.6%** | **5.3%** | **3%** | | **3.7%** | *45.8%* |
|  |  | total *E. coli* | | 2.6% | 3.2% | 7.9% | 18.2% | 13.2% | | 11.1% |  |
| Ante-  room | Boot swab  (n = 1) | Anteroom | Boot swab anteroom floor | - | - | - | - | - | | **6.7%**  13.3% | *60%* |
|  |  | floor |  |  |  |  |  |  |  |  |  |
|  | Gauze swab  (n = 21) | Anteroom | Drain hole | - | - | - | - | - | |  |  |
|  |  | floor | Floor cracks | - | - | -^#^ | - | +*^#^ | |  |  |
|  |  | Anteroom ceiling | Ceiling | - | - | - | - | - | | **0%**  0% | *0%* |
|  |  | Anteroom | Spider webs | - | - | - | - | - | | **0%**  0% | *10%* |
|  |  | wall | Lower wall | - | - | - | - | - | |  |  |
|  |  | Anteroom | Door outwards, lower part | - | -^#^ | - | -^#^ | -^#^ | | **0%**  16% | *44%* |
|  |  | door | Door to stable, lower part | - | - | - | -^#^ | - | |  |  |
|  |  |  | Door outwards, handle inside | - | - | - | - | - | |  |  |
|  |  |  | Door outwards, handle outside | - | - | - | - | - | |  |  |
|  |  |  | Door to stable, handle outside | - | - | - | - | - | |  |  |
|  |  | Anteroom | Sink’s drain | - | - | +*^#^ | - | -^#^ | | **1.8%**  9.1% | *25.5%* |
|  |  | interior | Dismantled metal boxes from stable | - | - | - | - | -^#^ | |  |  |
|  |  |  | Broom | - | - | - | - | - | |  |  |
|  |  |  | Trash bin lid | -^#^ | - | - | - | - | |  |  |
|  |  |  | Electrical devices | - | - | - | - | - | |  |  |
|  |  |  | Barrier for chickens separation | - | - | - | - | - | |  |  |
|  |  |  | Light switch | - | - | - | - | - | |  |  |
|  |  |  | Soap dispenser | - | - | - | - | - | |  |  |
|  |  |  | Weighing machine | - | - | - | - | - | |  |  |
|  |  |  | Rubber boots | - | - | - | - | -^#^ | |  |  |
|  |  |  | Water tap | - | - | - | - | - | |  |  |
|  | Rinse water  (n = 1) | Anteroom | Rinse water | - | - | - | - | - | | **0%**  0% | *0%* |
|  |  | rinse water |  |  |  |  |  |  |  |  |  |
|  | **Sum** | **ESBL-/pAmpC- *E. coli*** | | **0%** | **0%** | **4.4%** | **0%** | **4.4%** | | **1.7%** | *30.4%* |
|  |  | total *E. coli* | | 4.3% | 4.3% | 8.7% | 8.7% | 21.7% | | 9.6% |  |
| Surrounding  environment | Boot swab  (n = 4) | Surrounding | Supply air side | -^#^ | -^#^ | -^#^ | - | +*^#^ | | **35%**  75% | *100%* |
|  |  | floor | Exhaust air side | - | +°^#^ | -^#^ | -^#^ | +*^#^ | |  |  |
|  |  |  | Road A | - | -^#^ | -^#^ | -^#^ | +*^#^ | |  |  |
|  |  |  | Road B | - | +°^#^ | +*^#^ | - | +*^#^ | |  |  |
|  | Gauze swab  (n = 4) | Surrounding | Tractor tires (tractor for litter supply) | - | - | +*^#^ | - | -^#^ | | **10%**  30% | *100%* |
|  |  | tractor tires | Tractor tires (tractor for feed supply) | - | - | - | - | -^#^ | |  |  |
|  |  | Surrounding | Ventilation flap supply air | - | - | - | - | -^#^ | | **0%**  10% | *60%* |
|  |  | ventilation system | Ventilator | - | - | - | - | - | |  |  |
|  | **Sum** | **ESBL-/pAmpC- *E. coli*** | | **0%** | **25%** | **25%** | **0%** | **50%** | | **20%** | *90%* |
|  |  | total *E. coli* | | 12.5% | 50% | 75% | 25% | 87.5% | | 47.5% |  |
| **Total sum** | | **ESBL-/pAmpC- *E. coli*** | | **0%** | **7.2%** | **5.8%** | **2.9%** | **8.7%** | | **4.9%** | *45.8%* |
|  |  | total *E. coli* | | 4.3% | 13% | 14.5% | 15.9% | 24.6% | | 14.8% |  |

+ ESBL-/ pAmpC- *E. coli* detection, - no ESBL-/ pAmpC- *E. coli* detection; * detection of CTX-M-1, ° detection of CMY-2; ^#^ detection of non-selective *E. coli*, samples without reference showed no growth of *E. coli*; ^1^ Inner stables were structured in length (A-F) and width (1-3) into 18 sections for a reproducible sampling scheme (see Figure 1). Sampling locations with two samples were taken to cover the entire length of the barn (one sample in stable’s front part (I) and one sample in stable’s rear part (II)). For each position one gauze swab was used, e.g. sampling location “floor drain (C2)” = one gauze swab, sampling location “floor cracks I (A1, B2, C3)” = three gauze swabs

Supplementary Table 2: Characterization of ESBL- and pAmpC- E. coli detected before and after cleaning and disinfection (C&D) of the five investigated broiler stables (A - E).

| **Stable** | **Time-point** | **Sampling location** | **Isolate ID** | **PCR and PFGE results** | | | **WGS results** | | | |
| --- | --- | --- | --- | --- | --- | --- | --- | --- | --- | --- |
|  |  |  |  | Phylo-genetic group | ESBL-/  AmpC-  gene | XbaI- PFGE pattern | Serotype | Phylo-genetic group | MLST | Resistance determinants |
| A | 0 | Stable BS | ITU9492 | F | *bla*_CTX-M-1_ | I | n.d. | n.d. | n.d. | n.d. |
| B | 0 | Stable PF | ITU11074+ | A/C | *bla*_CMY-2_ | II | O86:H12 | A | 10 | *aadA1*, *bla*_CMY-2_, *bla*_TEM-1B_, *dfrA1*, *sul2* |
| B | 0 | Stable PF | ITU11075 | A/C | *bla*_CMY-2_ | II | n.d. | n.d. | n.d. | n.d. |
| B | 1 | Stable GS  Edge floor/wall II | ITU11133+ | A/C | *bla*_CMY-2_ | II | O86:H12 | A | 10 | *aadA1*, *bla*_CMY-2_, *bla*_TEM-1B_, *dfrA1*, *sul2* |
| B | 1 | Stable GS  Edge floor/wall II | ITU11134 | A/C | *bla*_CMY-2_ | II | n.d. | n.d. | n.d | n.d. |
| B | 1 | Stable GS  Edge floor/wall II | ITU11154 | A/C | *bla*_CMY-2_ | V | n.d. | n.d. | n.d | n.d. |
| B | 0 | Stable PF | ITU11078 | E/D | *bla*_CMY-2_ | VI | n.d. | n.d. | n.d | n.d. |
| B | 1 | Stable GS  Edge floor/wall II | ITU12012 | E/D | *bla*_CMY-2_ | IV | n.d. | n.d. | n.d | n.d. |
| B | 0 | Stable BS | ITU11087+ | F | *bla*_CMY-2_ | III | O132:H4 | G | 117 | *aadA1*, *bla*_CMY-2_, *bla*_TEM-1B_, *dfrA1*, *sul2* |
| B | 0 | Stable PF | ITU11082+ | F | *bla*_CMY-2_ | n.d. | O132:H4 | G | 117 | *aadA1*, *bla*_CMY-2_, *bla*_TEM-1B_, *dfrA1*, *sul2* |
| B | 1 | Surrounding BS  Road B | ITU11151+ | F | *bla*_CMY-2_ | III | O132:H4 | G | 117 | *aadA1*, *bla*_CMY-2_, *bla*_TEM-1B_, *dfrA1*, *sul2* |
| B | 1 | Surrounding BS  Exhaust air side | ITU11142+ | F | *bla*_CMY-2_ | IIIa | O132:H4 | G | 117 | *aadA1*, *bla*_CMY-2_, *bla*_TEM-1B_, *dfrA1*, *sul2* |
| C | 0 | Stable BS | ITU12042+ | F | *bla*_CTX-M-1_ | VII | Onovel12:H4 | G | 117 | *aadA1*, *bla*_CTX-M-1_, *bla*_TEM-1B_, *dfrA1*, *sul2* |
| C | 0 | Stable BS | ITU12045+ | F | *bla*_CTX-M-1_ | VIIb | Onovel12:H4 | G | 117 | *bla*_CTX-M-1_ |
| C | 1 | Stable GS  Edge floor/wall II | ITU12080+ | F | *bla*_CTX-M-1_ | VIIa | Onovel12:H4 | G | 117 | *aadA1*, *bla*_CTX-M-1_, *bla*_TEM-1B_, *dfrA1*, *sul2* |
| C | 1 | Anteroom GS  Sink’s drain | ITU12083+ | F | *bla*_CTX-M-1_ | VIIa | Onovel12:H4 | G | 117 | *aadA1*, *bla*_CTX-M-1_, *bla*_TEM-1B_, *dfrA1*, *sul2* |
| C | 1 | Surrounding GS  Tractor tires | ITU12089+ | F | *bla*_CTX-M-1_ | VIIc | Onovel12:H4 | G | 117 | *bla*_CTX-M-1_ |
| C | 1 | Surrounding BS  Road B | ITU12091+ | F | *bla*_CTX-M-1_ | VIIc | Onovel12:H4 | G | 117 | *bla*_CTX-M-1_ |
| D | 0 | Stable BS | ITU12950+ | F | *bla*_CTX-M-1_ | VIIIa | Onovel12:H4 | G | 117 | *bla*_CTX-M-1_ |
| D | 1 | Stable GS  Floor cracks | ITU12995+ | F | *bla*_CTX-M-1_ | VIIIa | Onovel12:H4 | G | 117 | *bla*_CTX-M-1_ |
| D | 1 | Stable GS  Edge floor/wall II | ITU13001+ | F | *bla*_CTX-M-1_ | VIII | Onovel12:H4 | G | 117 | *bla*_CTX-M-1_ |
| E | 0 | Stable BS | ITU13016^+^ | F | *bla*_CTX-M-1_ | IX | O143:H4 | G | 117 | *aph(3’’)-Ib*, *aph(3’)-Ia*, *aph(6)-Id*, *bla*_CTX-M-1_, *sul2*, *tet(B)* |
| E | 1 | Stable GS  Edge floor/wall II | ITU13921+ | F | *bla*_CTX-M-1_ | IX | O143:H4 | G | 117 | *aph(3’’)-Ib*, *aph(3’)-Ia*, *aph(6)-Id*, *bla*_CTX-M-1_, *sul2*, *tet(B)* |
| E | 1 | Anteroom GS  Floor cracks | ITU13927+ | F | *bla*_CTX-M-1_ | IX | O143:H4 | G | 117 | *aph(3’’)-Ib*, *aph(3’)-Ia*, *aph(6)-Id*, *bla*_CTX-M-1_, *sul2*, *tet(B)* |
| E | 1 | Surrounding BS  Exhaust air side | ITU13929+ | F | *bla*_CTX-M-1_ | IX | O143:H4 | G | 117 | *aph(3’’)-Ib*, *aph(3’)-Ia*, *aph(6)-Id*, *bla*_CTX-M-1_, *sul2*, *tet(B)* |
| E | 1 | Surrounding BS  Supply air side | ITU13933+ | F | *bla*_CTX-M-1_ | IXa | O143:H4 | G | 117 | *aph(3’’)-Ib*, *aph(3’)-Ia*, *aph(6)-Id*, *bla*_CTX-M-1_, *sul2*, *tet(B)* |
| E | 1 | Surrounding BS  Road A | ITU13937+ | F | *bla*_CTX-M-1_ | IXa | O143:H4 | G | 117 | *aph(3’’)-Ib*, *aph(3’)-Ia*, *aph(6)-Id*, *bla*_CTX-M-1_, *sul2*, *tet(B)* |
| E | 1 | Surrounding BS  Road B | ITU13939 | F | *bla*_CTX-M-1_ | X | n.d. | n.d. | n.d | n.d. |
|  |  |  |  |  |  |  |  |  |  |  |

0 = before C&D, 1 = after C&D; BS = boot swab, PF = pooled feces, GS = gauze swabs; ^+^ isolates investigated using whole genome sequencing (WGS); n.d. = not determined; one positive sampling location after C&D is not listed because there was no related isolate before C&D: stable B, wooden board at entry to stable (resistance gene *bla*_CMY-2_, phylogenetic group B1)

Supplementary Table 3: Detection of *Enterococci* on sampling locations in five investigated broiler stables (A-E) after cleaning and disinfection based on grouping into 14 sampling categories.

| **Sampling category**  **(number of sampled locations)** | **Stable** | ***Enterococci* detection (cfu/swab)** | | | |
| --- | --- | --- | --- | --- | --- |
|  |  | **negative** | **Enrichment** | **<10^7^** | **≥ 10^7^** |
|  |  |  |  |  |  |
| **Stable floor**  (n=7) | A | 5 | 1 | 1 | 0 |
|  | B | 2 | 1 | 0 | 4 |
|  | C | 2 | 1 | 2 | 2 |
|  | D | 1 | 1 | 2 | 3 |
|  | E | 1 | 2 | 3 | 1 |
| Sum (n=35) |  | 11 | 6 | 8 | 10 |
|  |  |  |  |  |  |
| **Stable wall**  (n=7) | A | 5 | 1 | 1 | 0 |
|  | B | 2 | 2 | 2 | 1 |
|  | C | 7 | 0 | 0 | 0 |
|  | D | 5 | 1 | 1 | 0 |
|  | E | 4 | 2 | 1 | 0 |
| Sum (n=35) |  | 23 | 6 | 5 | 1 |
|  |  |  |  |  |  |
| **Stable ceiling**  (n=6) | A | 4 | 2 | 0 | 0 |
|  | B | 3 | 3 | 0 | 0 |
|  | C | 6 | 0 | 0 | 0 |
|  | D | 2 | 2 | 2 | 0 |
|  | E | 5 | 0 | 1 | 0 |
| Sum (n=30) |  | 20 | 7 | 3 | 0 |
|  |  |  |  |  |  |
| **Stable interior**  (n=15) | A | 7 | 2 | 5 | 1 |
|  | B | 9 | 1 | 2 | 3 |
|  | C | 11 | 4 | 0 | 0 |
|  | D | 7 | 3 | 5 | 0 |
|  | E | 9 | 1 | 5 | 0 |
| Sum (n=75) |  | 43 | 11 | 17 | 4 |
|  |  |  |  |  |  |
| **Stable ventilation system**  (n=3) | A | 1 | 0 | 2 | 0 |
|  | B | 1 | 0 | 1 | 1 |
|  | C | 2 | 0 | 1 | 0 |
|  | D | 1 | 2 | 0 | 0 |
|  | E | 1 | 1 | 0 | 1 |
| Sum (n=15) |  | 6 | 3 | 4 | 2 |
|  |  |  |  |  |  |
| **Anteroom floor**  (n=3) | A | 2 | 1 | 0 | 0 |
|  | B | 2 | 1 | 0 | 0 |
|  | C | 0 | 0 | 3 | 0 |
|  | D | 1 | 1 | 1 | 0 |
|  | E | 1 | 0 | 2 | 0 |
| Sum (n=15) |  | 6 | 3 | 6 | 0 |
|  |  |  |  |  |  |
| **Surrounding ventilation system**  (n=2) | A | 1 | 0 | 1 | 0 |
|  | B | 2 | 0 | 0 | 0 |
|  | C | 1 | 0 | 1 | 0 |
|  | D | 0 | 2 | 0 | 0 |
|  | E | 0 | 0 | 2 | 0 |
| Sum (n=10) |  | 4 | 2 | 4 | 0 |
|  |  |  |  |  |  |
| **Surrounding tractor tires**  (n=2) | A | 0 | 0 | 2 | 0 |
|  | B | 0 | 1 | 0 | 1 |
|  | C | 0 | 0 | 1 | 1 |
|  | D | 0 | 0 | 2 | 0 |
|  | E | 0 | 0 | 1 | 1 |
| Sum (n=10) |  | 0 | 1 | 6 | 3 |
|  |  |  |  |  |  |
| **Surrounding floor**  (n=4) | A | 0 | 1 | 2 | 1 |
|  | B | 0 | 0 | 0 | 4 |
|  | C | 0 | 0 | 2 | 2 |
|  | D | 0 | 0 | 1 | 3 |
|  | E | 0 | 0 | 0 | 4 |
| Sum (n=20) |  | 0 | 1 | 5 | 14 |
|  |  |  |  |  |  |
| **Anteroom ceiling**  (n=1) | A | 1 | 0 | 0 | 0 |
|  | B | 1 | 0 | 0 | 0 |
|  | C | 1 | 0 | 0 | 0 |
|  | D | 1 | 0 | 0 | 0 |
|  | E | 1 | 0 | 0 | 0 |
| Sum (n=5) |  | 5 | 0 | 0 | 0 |
|  |  |  |  |  |  |
| **Anteroom wall**  (n=2) | A | 2 | 0 | 0 | 0 |
|  | B | 1 | 0 | 0 | 1 |
|  | C | 2 | 0 | 0 | 0 |
|  | D | 2 | 0 | 0 | 0 |
|  | E | 2 | 0 | 0 | 0 |
| Sum (n=10) |  | 9 | 0 | 0 | 1 |
|  |  |  |  |  |  |
| **Anteroom door**  (n=5) | A | 3 | 1 | 1 | 0 |
|  | B | 3 | 1 | 0 | 1 |
|  | C | 4 | 0 | 1 | 0 |
|  | D | 2 | 1 | 2 | 0 |
|  | E | 2 | 2 | 0 | 1 |
| Sum (n=25) |  | 14 | 5 | 4 | 2 |
|  |  |  |  |  |  |
| **Anteroom rinse water**  (n=1) | A | 1 | 0 | 0 | 0 |
|  | B | 1 | 0 | 0 | 0 |
|  | C | 1 | 0 | 0 | 0 |
|  | D | 1 | 0 | 0 | 0 |
|  | E | 1 | 0 | 0 | 0 |
| Sum (n=5) |  | 5 | 0 | 0 | 0 |
|  |  |  |  |  |  |
| **Anteroom interior**  (n=11) | A | 7 | 1 | 3 | 0 |
|  | B | 11 | 0 | 0 | 0 |
|  | C | 9 | 1 | 1 | 0 |
|  | D | 7 | 4 | 0 | 0 |
|  | E | 7 | 0 | 3 | 1 |
| Sum (n=55) |  | 41 | 6 | 7 | 1 |
|  |  |  |  |  |  |

Information on sampling locations is given in Supplementary Table 1; negative = no detection of *Enterococci*; Enrichment = qualitative detection after enrichment; cfu = colony forming units
